# Detection of incipient rotor unbalance fault based on the RIME-VMD and modified-WKN

**DOI:** 10.1038/s41598-024-54984-z

**Published:** 2024-02-26

**Authors:** Qian Wang, Shuo Hu, Xinya Wang

**Affiliations:** 1 College of Electrical Information Engineering, Zhengzhou University of Light Industry, Zhengzhou, 450000 China; 2 IoT Equipment Research Institute, GL TECH Co., Ltd., Zhengzhou, 450000 China

**Keywords:** Energy infrastructure, Mechanical engineering, Energy infrastructure, Mechanical engineering

## Abstract

Due to the high incidence and inconspicuous initial characteristics of rotor unbalance faults, the detection of incipient unbalance faults is becoming a very challenging problem. In this paper, a new method of small rotor unbalance fault diagnosis based on RIME-VMD and modified wavelet kernel network (modified-WKN) is proposed. Firstly, in order to extract the small unbalance fault information from the vibration signals with low signal-to-noise ratio (SNR) more efficiently, the RIME algorithm is used to search for the optimal location of the penalty factor and decomposition layer in the variable mode decomposition (VMD). Secondly, the most relevant decomposition components to the small unbalance fault information are selected by using Pearson Correlation Coefficients and utilized to reconstruct the signal. Finally, the modified-WKN diagnostic model that is used for multi-sensor data fusion is constructed. The model can acquire features of vibration signals from multiple position sensors, which enhances the ability of the modified WKN diagnostic model to deal with incipient fault modes. Based on the experimental analysis of rotor unbalance fault datasets with different SNRs, it is verified that the detection performance of the proposed method is better than the traditional WKN and VMD-WKN methods. Specifically, the proposed method is more sensitive to the initial unbalance faults.

## Introduction

As the core component of rotating machinery, the rotor system has been extensively applied in the aerospace, petrochemical, coal, and electricity industries^1,2^. The primary faults in the rotor system include rotor unbalance, misalignment, rub-impact, and others^3^. Among these, rotor unbalance is a significant cause of instability in rotor systems. In practical engineering, the early characteristics of rotor unbalance fault signals are relatively weak. Additionally, they are always accompanied by noise and other uncertainties, leading to even weaker features. Therefore, the rapid and accurate detection of initial rotor unbalance faults is a very challenging diagnostic problem and is also a crucial safeguard for the long-term safe and stable operation of rotating machinery systems.

In the past few decades, the diagnosis methods for rotor faults can be divided into two categories^4^: one is time-frequency fault diagnosis methods, such as the wavelet transform, variational modal decomposition (VMD), and others^5–7^. The other is knowledge-based fault diagnostic methods, which includes support vector machines, expert systems, dynamic learning, and deep learning^8–11^. Currently, deep learning-based fault diagnosis methods have become a research hotspot, and various advanced learning models (CNN, LSTM, DBN, AE, and others) are widely utilized in the field of rotor fault diagnosis^12–17^.

Among these deep learning models, CNNs stand out for their exceptional performance in fault diagnosis^18^. However, the CNN-based models are often considered as black boxes due to the lack of interpretability. With the advancement of learning methods, various approaches have been proposed to enhance interpretability. With the advancement of learning methods, various approaches have been proposed to enhance interpretability. Zilke et al.^19^ developed a novel scheme for neural network rule extraction based on decision trees to investigate the decision-making process. Grezmak et al.^20^ utilized layer-by-layer correlation propagation as an indicator to elucidate the key features learning process of CNN from time-frequency spectrum images. Jia^21^ employed a Neuron Activation Maximization algorithm to visualize the kernels of convolutional layers, aiming to comprehend the process of feature learning. Chen^22^ applied Gradient Class Activation Mapping to generate an attention model and explained the model by analyzing attention matters. Li et al.^23^ introduced an attention mechanism to assist deep neural networks in focusing on critical data segments, and the learned fault diagnosis characteristics can be presented in a visualized manner.

It should be noted that the rotor fault diagnosis signals are vibration data, and the above-mentioned methods are mainly suitable for processing two-dimensional image data. In order to solve this problem, Li et al.^24^ proposed an interpretable model known as the Wavelet-Kernel Network (WKN), which is suitable for dealing with the vibration fault signals. The wavelet transformation is employed in the first convolutional layer of CNN, and the physical significance of wavelet transformation is taken as the interpretability process. However, WKN is implemented based on a single sensor signal and cannot fully capture the fault information concealed within the noise in the rotor vibration signals.

Inspired by the WKN method, this paper proposes a new rotor unbalance fault diagnosis method based on a RIME-VMD and modified-WKN. Firstly, to extract the initial unbalance fault information accurately under the condition of complex noises, the VMD decomposition algorithm is employed to decompose the vibration signals. In addition, the RIME algorithm^25^ is used to search for the optimal combination of penalty factor $\alpha$ and decomposition layer *k* of VMD. Secondly, the obtained optimal IMF components are selected by using the Pearson Correlation Coefficient (PCC), and the most relevant fault IMF components are used for the signal reconstruction. Thirdly, a new multi-head convolutional layer of the WKN is constructed to capture rotor unbalance fault information comprehensively based on the multiple vibration data from different positions in the rotor system. Additionally, this paper adopts multi-scale convolution to extract fault information of various scales in the fused features, which enhances the ability to perceive complex patterns. Finally, the diagnostic performance of the proposed method is illustrated based on the experimental analysis with varying SNRs. The results demonstrate that it is better than the traditional WKN method and the WKN combined with VMD (VMD-WKN) methods. Specifically, the proposed method is more sensitive to the initial unbalance faults.

The main contributions of this article are shown as follows:

1. The most relevant unbalance fault IMF components are obtained according to the parameter-optimized VMD method, and the optimal combination of penalty factor $\alpha$ and number of decomposition layers *k* is automatically searched by embedding the RIME algorithm.
2. Different from the WKN in Ref.^24^, the modified-WKN fault diagnosis model was constructed by fully considering vibration data from different positions. The rotor unbalance fault information from the rotor vibration signals concealed within the noises are fully captured by using the multi-head convolution and multi-scale convolution structures. Compared with WKN using single sensor data, the modified-WKN model exhibits greater sensitivity to the small initial unbalance fault information.

The remaining sections of this article are structured as follows. "Theoretical basis" section covers the theoretical basis, introducing the theoretical foundation of the RIME algorithm and WKN. "Proposed method" section introduces the structures of the parameter-optimized VMD and modified-WKN models. "Data acquisition" section presents the rotor fault test bench and unbalance datasets. "Experimental results" section covers the experiments, and the conclusion presented in "Conclusion" section.

## Theoretical basis

### RIME optimization algorithm

The Rime algorithm^25^ was proposed by Huang in 2023, which is an intelligent search method by simulating the growth process of rime in nature. The Rime algorithm can be divided into the following four phases.

In the first stage, the entire rime population *R* is initialized. The parameter *R* can be expressed by the following equation:

1 $$\begin{aligned} R=\left( \begin{array}{c} S_{1} \\ S_{2} \\ \vdots \\ S_{i} \end{array}\right) ; S_{i}=\left[ \begin{array}{llll} x_{i 1}&x_{i 2}&\cdots&x_{i j} \end{array}\right] \end{aligned}$$

where $S_{i}$ represents the *ith* rime agent; $x_{ij}$ denotes the *jth* rime particle within this agent.

The second stage is called the Soft-rime Search mechanism. The mechanism simulates the random diffusion and large area coverage of rime particles in a weak wind environment. The updated position of rime particles $R_{ij}^{\text {new}}$ in a weak wind environment can be expressed by the following equation:

2 $$\begin{aligned} R_{ij}^{\text {new}} = R_{\text {best}, j} + r_{1} \cdot \cos \varphi \cdot \beta \cdot \left( h\left( Ub_{ij} - Lb_{ij}\right) + L b_{ij}\right) , \quad r_{2} < E \end{aligned}$$

where $R_{i j}^{\text{ new }}$ is the updated position of the rime particle, $R_{\text{ best,j }}$ is the *jth* particle of the best rime agent in the population *R*. A random number *h* is used to control the center distance between two rime particles, which has the value in the range of (0, 1). The parameter $Ub_{ij}$ and the parameter $Lb_{ij}$ are the top and bottom bounds of the escape space, respectively. The movement direction of rime particles is influenced by the random variable $r_{1}$, where $r_{1} \in (0,1)$. Both $r_{1}$ and $\cos \varphi$ vary with the iteration count. The $\varphi$ is an angle over time that is affected by the current number of iterations *t* and the maximum number of iterations of the algorithm *T*. The mathematical expression for $\varphi$ is as follows:

3 $$\begin{aligned} \varphi = \pi \cdot \frac{t}{10 T} \end{aligned}$$

The mathematical model for the environmental factor $\beta$ is a step function. In the Soft-rime search strategy, $\beta$ is utilized to simulate the impact of the external environment. The mathematical expression for $\beta$ is given by:

4 $$\begin{aligned} \beta = 1 - \left[\frac{\omega \cdot t}{T} \right] / {\omega } \end{aligned}$$

where the parameter $[\cdot ]$ indicates rounding; the default setting of the parameter $\omega$ is 5, which is used to regulate the number of segments of the step function.

Parameter *E* represents the coefficient of being attached; $r_{2}$ is a random number. The *E* controls particle position update along with $r_{2}$. The range of values for $r_{2}$ is detailed in the Ref.^26^. The *E* can be expressed with the following equation:

5 $$\begin{aligned} {E=\sqrt{(t / T)}} \end{aligned}$$

The third stage is known as the Hard-rime puncture mechanism. It promotes the exchange of information between ordinary agents and optimal agents by stimulating the growth of rime in strong wind conditions, thereby improving the precision of algorithmic solutions. The replacement equation for the particle position in the strong wind condition can be expressed by the following equation:

6 $$\begin{aligned} R_{ij}^{\text {new}} = R_{\text {best}, j}, \quad r_{3} < F^{\text {normr}}\left( S_{i}\right) \end{aligned}$$

where $r_{3}$ is a random number with a range of (-1,1); $F^{\text{ normr }}\left( S_{i}\right)$ is the normalized fitness value.

The fourth stage is called the Positive Greedy Selection Mechanism. If the updated fitness value is superior to the previous value, the agent’s fitness value and solution are replaced.

### Theoretical basis of WKN

The Wavelet transform is a time-frequency analysis method that includes Continuous Wavelet Transform (CWT) and Discrete Wavelet Transform (DWT). Different from the Fourier transform, the basis function of the wavelet transform is a wavelet basis with finite length and attenuation. The Continuous Wavelet Convolution((CWConv) layer is implemented by utilizing the similarity between CNN convolution operations and CWT operations.

The convolution operation performed by the convolution kernel on the input signal when it passes through the convolutional layer of the CNN can be considered as an inner product operation. This process can be represented by the following equation:

7 $$\begin{aligned} h = W \otimes x + b \end{aligned}$$

where *x* represents the current input data; *h* is denoted as the feature map obtained after convolutional computation; $\otimes$ denotes the convolution operator; *W* stands for the convolutional kernel weight; *b* represents the bias.

Similarly, the process of CWT can be viewed as the inner product operation between the input signal and the wavelet basis functions. The continuous wavelet transform of the signal $X(t)$ can be expressed as follows:

8 $$\begin{aligned} {\text {CWT}}_{f}(u, s)=\left\langle X, \psi _{u, s}(t)\right\rangle =\frac{1}{\sqrt{s}} \int X(t) \psi ^{*}\left( \frac{t-u}{s}\right) dt \end{aligned}$$

where $s$ is the scale parameter; $u$ is the translation parameter; $t$ is the time parameter; $x(t)$ is the input signal; $\psi ^{*}$ is the complex conjugate of the wavelet basis function $\psi _{\textrm{u}, \textrm{s}}$. The wavelet basis functions $\psi _{\textrm{u}, \textrm{s}}$ can be expressed by the following equation:

9 $$\begin{aligned} \psi _{u, s}(t)=\frac{1}{\sqrt{s}} \psi \left( \frac{t-u}{s}\right) \end{aligned}$$

In summary, the CWConv layer was designed based on the principle of CWT. Subsequently, the first convolutional layer of the CNN was replaced with CWConv to construct the WKN. The CWConv layer was designed to introduce interpretability to the model. The convolution operation performed by the CWConv layer on the input signal can be expressed by the following equation:

10 $$\begin{aligned} H=\psi _{u, {~s}}({t}) * {~g}({x}) \end{aligned}$$

where $H$ represents the feature values output by the CWConv layer; $g(x)$ represents the input signal; $*$ denotes the convolution operation.

**Figure 1:**
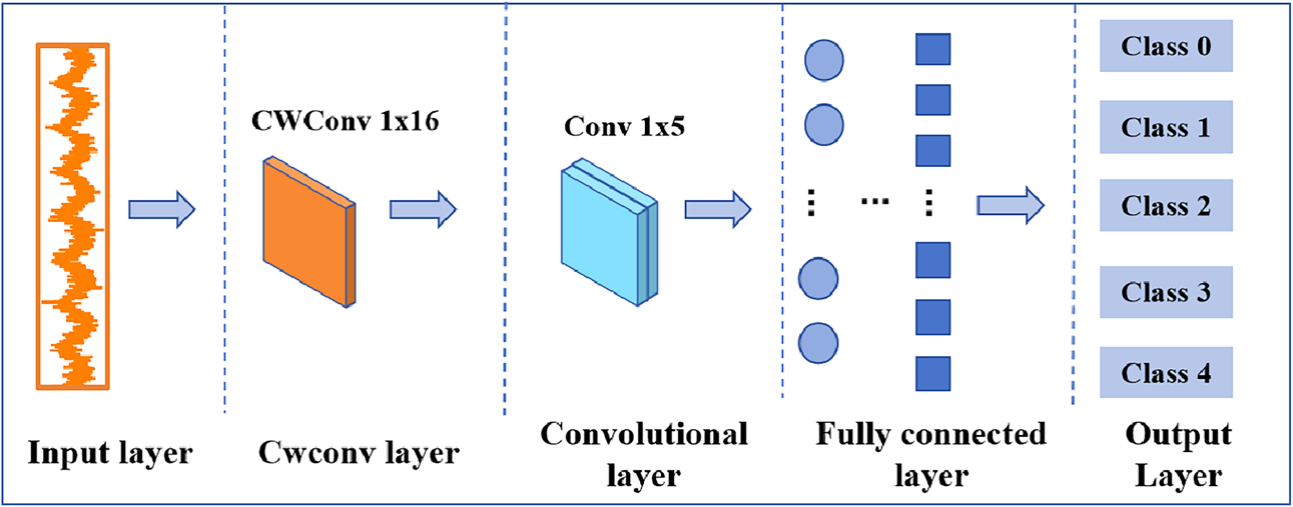
WKN structure.

The core part of the WKN is the selection of the wavelet kernel basis functions in the CWConv layer. In Ref. ^24^, it has been proved that the Laplace wavelet employed in the WKN has the best performance in rotating machinery fault diagnosis. The structure of WKN is illustrated in Fig. 1, comprising the input layer, continuous wavelet convolutional layer, convolutional layer, fully connected layer, and output layer.

## Proposed method

### RIME-VMD

The VMD is a signal decomposition method with adaptive features. The detailed decomposition composition process of VMD can be found in Ref.^27^. The implementation process of the VMD algorithm can be regarded as the solution process of the variational problem. The description of the constrained variational model is as follows:

11 $$\begin{aligned} \left\{ \begin{array}{l} \min _{u_k,\omega _k} \left\{ \sum _{k=1}^K{\left\| \partial _t\left[ \left( \delta (t)+\frac{j}{\pi t} \right) \times u_k(t) \right] e^{-j\omega _kt} \right\| _{2}^{2}} \right\} \\ \,\,\textrm{s}.\textrm{t}.\sum _{k=1}^K{u_k}(t)=f(t)\\ \end{array} \right. \end{aligned}$$

where $\partial _{t}$ represents the partial derivative with respect to ${t}; \delta (t)$ is the impulse function; $u_{k}(t)$ is the *kth* mode function; $\omega _{k}$ is the central frequency of the *kth* mode; *f*(*t*) is the original signal.

In order to solve the optimal solution of Equation (11), the quadratic penalty factor $\alpha$ and the Lagrange operator equation are introduced to transform the constrained variational problem into an unconstrained variational problem. The augmented Lagrangian quantity *L* can be expressed by the following equation:

12 $$\begin{aligned} \begin{aligned} \begin{array}{c} L\left( \left\{ \mu _{k}\right\} ,\left\{ \omega _{k}\right\} , \lambda \right) =\alpha \sum _{k}\left\| \partial _{t}\left[ \left( \delta (t)+\frac{j}{\pi t}\right) \mu _{k}(t)\right] e^{-j \omega _{k} t}\right\| _{2}^{2} \\ \quad +\left\| f(t)-\sum _{k} \mu _{k}(t)\right\| _{2}^{2}+\left\langle \lambda (t), f(t)-\sum _{k} \mu _{k}(t)\right\rangle \ \end{array} \end{aligned} \end{aligned}$$

where ${L}(\cdot )$ represents the augmented Lagrangian function; $\alpha$ is the penalty factor; $\lambda (t)$ is the Lagrange multiplier.

The method of alternate multiplication is used in order to obtain the optimal solution of Equation (12). The update equation for $u_{k}$ and $\omega _{k}$are as follows:

13 $$\begin{aligned} {\hat{u}}_{k}^{n_{1}+1}(\omega )=\frac{{\hat{f}}(\omega )-\sum _{i \ne k} {\hat{u}}_{i}(\omega )+{\hat{\lambda }}(\omega ) / 2}{1+2 \alpha \left( \omega -\omega _{k}\right) ^{2}} \end{aligned}$$

14 $$\begin{aligned} \omega _{k}^{n_{1}+1}=\frac{\int _{0}^{\infty } \omega \left| {\hat{u}}_{k}(\omega )\right| ^{2} \mathrm {~d} \omega }{\int _{0}^{\infty }\left| {\hat{u}}_{k}(\omega )\right| ^{2} \mathrm {~d} \omega } \end{aligned}$$

where $n_{1}$ represents the iteration number; ${\hat{u}}_{k}^{n_{1}+1}(\omega )$, ${\hat{f}}(\omega )$, ${\hat{u}}_{k}(\omega )$, and ${\hat{\lambda }}(\omega )$ are the Fourier transforms of $u_{k}^{n_{1}+1}(t)$, *f*(*t*), $u_{k}(t)$, and $\lambda (t)$, respectively.

When processing signals with the VMD algorithm, two key parameters need to be preset, namely, the penalty factor $\alpha$ and the number of decomposition layers *K*. Moreover, the VMD decomposition results are greatly influenced by these two key parameters^28^. Therefore, selecting an appropriate combination of parameters is the key to processing signals using the VMD algorithm. In practical work, the values of *K* and $\alpha$ are typically estimated based on experience. However, due to the complexity of real signals, estimating the parameter values only empirically will not obtain optimal decomposition results. This will result in the inability to accurately extract weak incipient unbalance fault features from low signal-to-noise ratio signals. Therefore, the RIME algorithm is used in this paper to search for the optimal values of the parameter combinations *K* and $\alpha$. After the optimal values of *K* and $\alpha$ are obtained, the VMD method is then used to process the signal. Finally, the obtained optimal IMF components are selected by using the PCC, and the most relevant fault IMF components are used for the signal reconstruction.

The magnitude of the envelope entropy $E_{P}$ can reflect the sparsity property of the IMF component. When the decomposed IMF component contains more noise, the sparsity of this IMF component is weak, and the corresponding $E_{P}$ is larger. On the contrary, if the IMF component contains regular fault shocks, then the IMF component has strong sparsity, and the corresponding envelope has smaller entropy. Therefore, in this paper, the $E_{P}$ is chosen as the fitness function. The envelope entropy of the signal *x*(*j*) can be expressed by the following equation:

15 $$\begin{aligned} \left\{ \begin{array}{l} E_{P}=-\sum _{j=1}^{N} p_{j} \lg p_{j} \\ p_{j}=a(j) / \sum _{j=1}^{N} a(j) \end{array}\right. \end{aligned}$$

where *N* represents the length of the signal *x*(*j*), $p_{j}$ is the normalized form of *a*(*j*); *a*(*j*) is the envelope signal obtained by Hilbert demodulation of the signal *x*(*j*).

**Algorithm 1:**
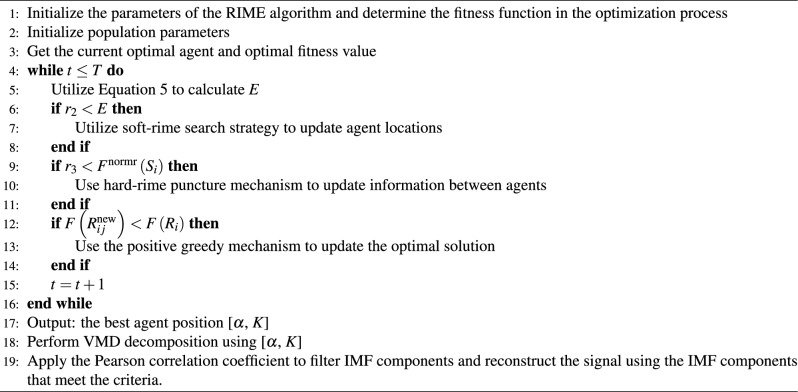
Pseudo-code of RIME-VMD.

### Modified-WKN

At present, rotating machinery is rapidly developing in the direction of intelligence, large-scale and complexity. However, due to the harsh operating environment and noise, it is difficult to fully capture the operating status of equipment with data from a single sensor^29–31^. Therefore, in this paper, multiple sensors are utilized to collect vibration data of the rotor system from different directions (the sensor arrangement will be described in the "Data acquisition" section), and the modified-WKN for multi-source data fusion is constructed. The information from sensors at different locations is fused through a multi-head CWConv layer. Then, the fault information at different scales in multi-source features is captured by multi-scale convolution.

**Figure 2:**
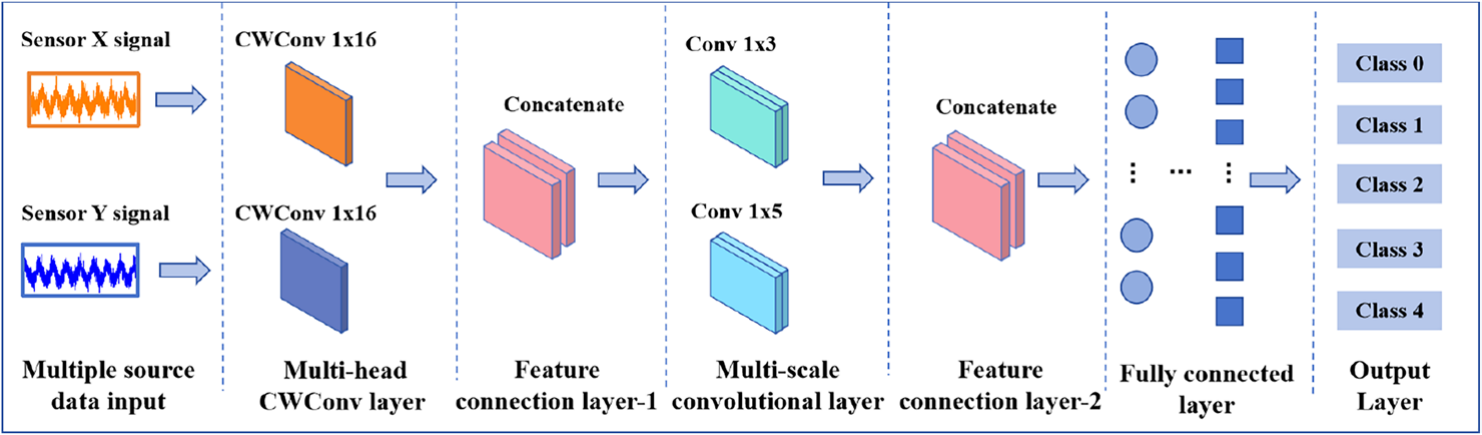
Modified-WKN structure.

The structure of the modified-WKN proposed in this paper is shown in Fig. 2, which includes a multi-data source input layer, a multi-head CWConv layer, a multi-scale convolutional layer, the feature connection layer, a fully connected layer, and an output layer. Different from the WKN structure as shown in Fig. 1, the multi-head wavelet convolution layer is composed of two CWConv layers (with a convolution kernel size of $1 \times 16$) that use Laplace wavelets. The multi-scale convolution layer is composed of two convolution kernels of different sizes. The output features of the multi-head CWConv layer are connected together by Concat and input to the multi-scale convolutional layer.

### Fault diagnosis method based on RIME-VMD and modified-WKN

**Figure 3:**
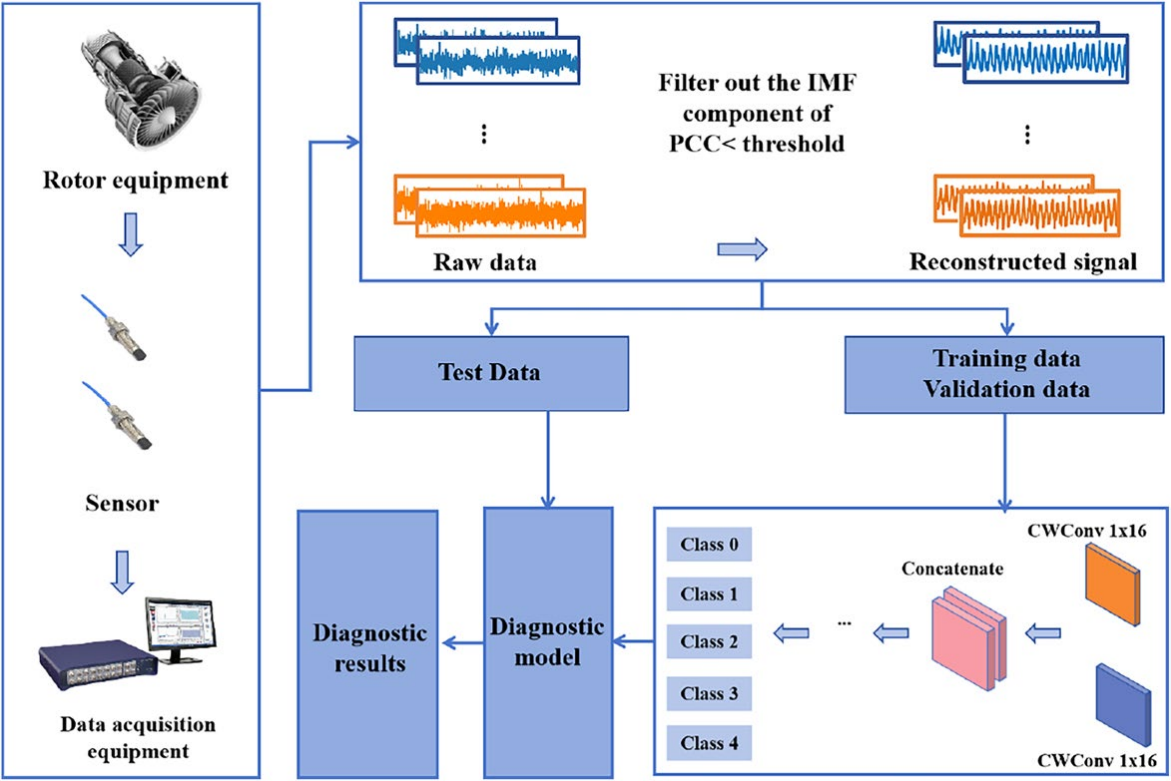
Diagnosis pipeline based on RIME-VMD and modified-WKN.

The fault diagnosis process based on the RIME-VMD and modified-WKN proposed in this article is shown in Fig. 3. (1) The vibration data for the different directions of the rotor system is collected by sensors in several positions. (2) The collected vibration data of the rotor system in different directions are decomposed by the RIME-VMD. Then, the PCC is utilized to select the optimal IMF components which are rich in fault information. Finally, the selected optimal IMF components are used for signal reconstruction. (3) The reconstructed dataset is divided into the training set, the validation set, and the test set. (4) The model is trained with the training set and the validation set. The model is saved after training is completed. (5) The accuracy and generalization ability of the model was evaluated with a test set.

## Data acquisition

**Figure 4:**
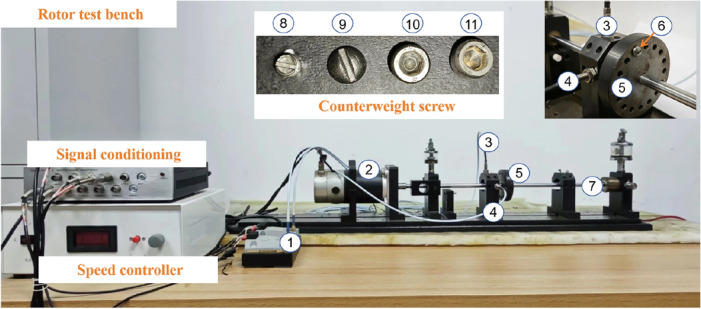
Rotor test bench. $\textcircled {1}$ Premplifier, $\textcircled {2}$ Rotor Motor, $\textcircled {3}$ Eddy current sensor (vertical direction), $\textcircled {4}$ Eddy current sensor (horizontal direction), $\textcircled {5}$ Rotor Shaft, $\textcircled {6}$ Counterweight screw, $\textcircled {7}$ Bearing latex. $\textcircled {8}$ 1.2 g counterweight screw $\textcircled {9}$ 2.5 g counterweight screw $\textcircled {10}$ 3.6 g counterweight screw $\textcircled {11}$ 5.0 g counterweight screw.

The rotor unbalance experiment was conducted on a rotor system test bench. As shown in Fig. 4, the test bench consists of a motor, a motor speed control device, a signal conditioning device, the eddy current sensor, and an unbalance device. The eddy current sensors are located in the horizontal (Y direction) and vertical (X direction) directions, respectively. In this paper, different weights of counterweight screws are added to the unbalance device to simulate four degrees of unbalance faults.

In industrial production, the motors are usually operated at multiple rotational speeds to adapt to different work conditions and production scenarios. Therefore, in this paper, the motor speeds are set as 1200 rpm, 1600 rpm, and 1800 rpm to simulate the real rotor work condition. The sampling frequency was set as 5120 Hz, and the rotor fault test bench saved data every 2 s. In this paper, four different degrees of unbalance faults are preset on the rotor system fault test bench, namely: incipient unbalance (1.2 g counterweight), mild unbalance (2.5 g counterweight), moderate unbalance (3.6 g counterweight), and severe unbalance (5.0 g counterweight). In addition, the normal state (0 g counterweight) is also included, so there are five operating states in total. A total of 300 signal samples were collected for each condition, and the length of each signal sample was 10240. Therefore, the total number of samples in the rotor unbalance fault dataset is 1500 files, which contain different rotational speeds and counterweights, as shown in Table 1. The training set, validation set, and test set were divided as 6:2:2. Thus, the training set contains 900 training samples, and the test and validation set each contains 300 samples. The incipient unbalance fault in this paper refers to a rotor system that has just experienced an unbalance fault. At this stage, the signal characteristics are very similar to those under normal conditions, and these features are easily overwhelmed by noise^32,33^.

**Figure 5:**
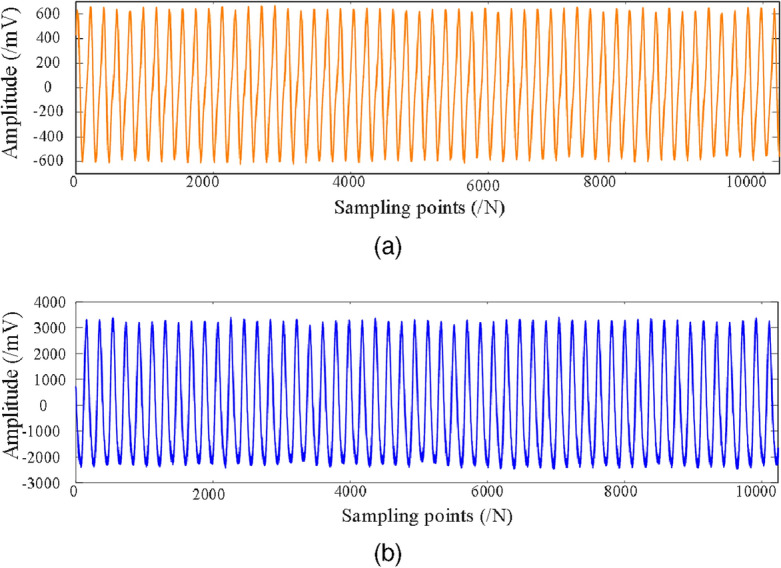
The original data collected from the test bench. (**a**) The original signal in the X direction. (**b**) The original signal in the Y direction. .

**Figure 6:**
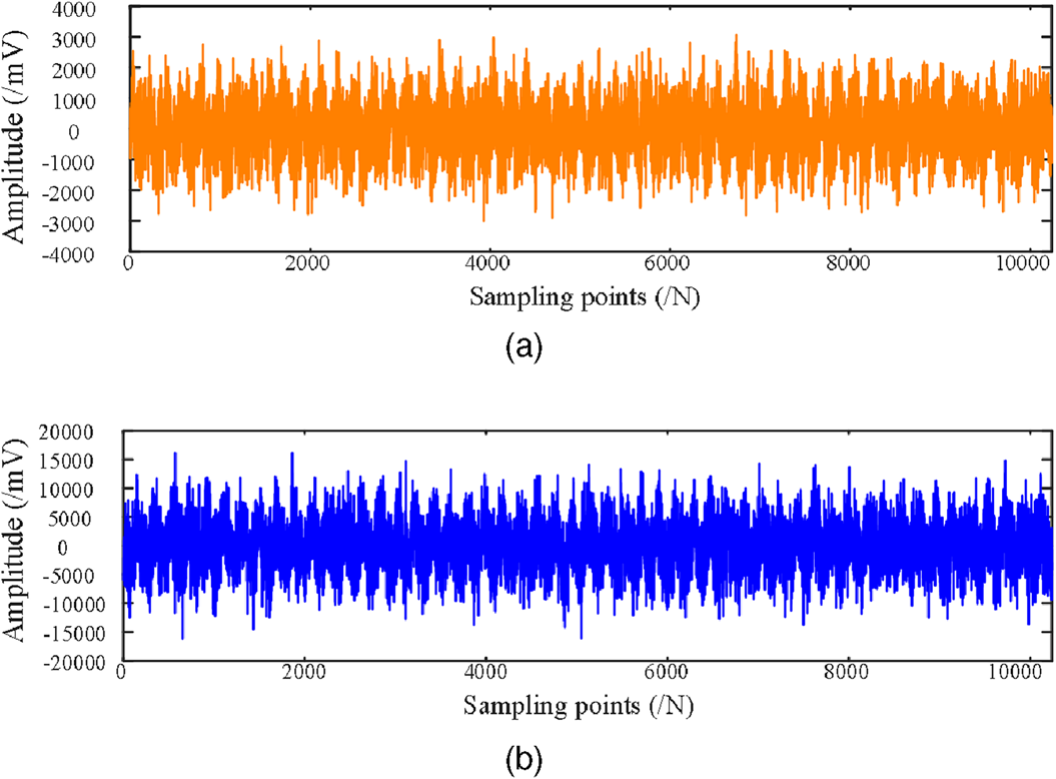
The original data from the test bench with added $-6$ dB noise. (**a**) The X direction signal with added $-6$ dB noise. (**b**) The Y direction signal with added $-6$ dB noise .

In real industrial production environments, the collected data are often mixed with a large amount of unavoidable noise. However, the unbalance fault dataset collected on the rotor test bed contains less disturbing components, which are not representative of the real industrial environment. In order to make the test results realistic, this paper adds different levels of Gaussian noise (0 dB, $-2$ dB, $-4$ dB, $-6$ dB) to the original dataset to simulate the real industrial environment. The original vibration data of the incipient unbalance fault associated with the 1.2 g counterweight at 1600 rpm is shown in Fig. 5, where Fig. 5a shows the X direction, and Figure  5b shows the Y direction. The data with $-6$ dB noise added is shown in Fig. 6. The Fig. 6a shows the time-waveform from the X direction. The Fig. 6b shows the time-waveform from the Y direction.

**Table 1:** Rotor speeds and counterweights.

Rotor speeds	Weight 0 g	Weight 1.2 g	Weight 2.5 g	Weight 3.6 g	Weight 5.0 g
1200 rpm	100	100	100	100	100
1600 rpm	100	100	100	100	100
1800 rpm	100	100	100	100	100

## Experimental results

### Results based on the modified-WKN

In order to verify the superiority of the proposed modified-WKN model, the WKN-X (vertical direction data diagnostic model), WKN-Y (horizontal direction data diagnostic model), and modified-WKN were separately trained on the dataset with added Gaussian white noise. The WKN (WKN- X, WKN- Y) structure is shown in Table 2, and the modified-WKN structure is shown in Table 3.

**Table 2:** WKN structural parameters.

Layer	Kernel size	Stride	Output size
CwConv	$1\times 16$	$1\times 1$	$1\times 64\times 10227$
MaxPool1d	$1\times 2$	$1\times 2$	$1\times 64\times 5133$
Conv2	$1\times 3$	$1\times 1$	$1\times 64\times 5111$
MaxPool1d	$1\times 2$	$1\times 2$	$1\times 64\times 2555$
Conv3	$1\times 5$	$1\times 1$	$1\times 16\times 2551$
AdaptiveMaxPool1d	-	-	$1\times 16\times 25$
Linear-1	-	-	$1\times 120$
Linear-2	-	-	$1\times 84$
Linear-3	-	-	$1\times 5$

**Table 3:** Modified-WKN structural parameters.

Layer	Kernel size	Stride	Output size
CwConv-X	$1 \times 16$	$1 \times 1$	$1 \times 64 \times 10227$
MaxPool	$1 \times 2$	$1 \times 2$	$1 \times 64 \times 5113$
CwConv-Y	$1 \times 16$	$1 \times 1$	$1 \times 64 \times 10227$
MaxPool1d	$1 \times 2$	$1 \times 2$	$1 \times 64 \times 5113$
Concat	-	-	$1 \times 128 \times 5113$
Conv3-1	$1 \times 3$	$1 \times 1$	$1 \times 64 \times 5111$
MaxPool	$1 \times 2$	$1 \times 2$	$1 \times 64 \times 2555$
Conv3-2	$1 \times 3$	$1 \times 1$	$1 \times 16 \times 2553$
AdaptiveMaxPool1d	–	–	$1 \times 16 \times 25$
Conv5-1	$1 \times 5$	$1 \times 1$	$1 \times 64 \times 5109$
MaxPool	$1 \times 2$	$1 \times 2$	$1 \times 64 \times 2554$
Conv5-2	$1 \times 5$	$1 \times 1$	$1 \times 16 \times 2500$
AdaptiveMaxPool1d	-	-	$1 \times 16 \times 25$
Concat	–	–	$1 \times 32 \times 25$
Linear-1	–	–	$1 \times 120$
Linear-2	–	–	$1 \times 84$
Linear-3	–	–	$1 \times 5$

**Figure 7:**
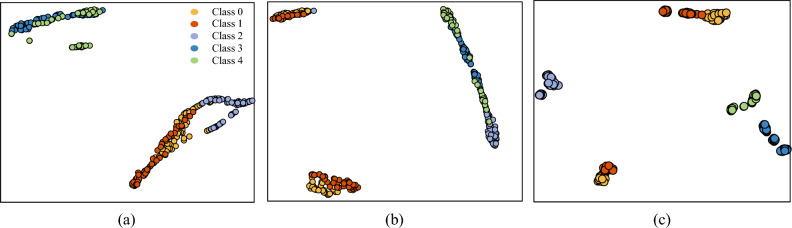
t-SNE visualization of the WKN-X, WKN-Y, and modified-WKN classification results on the $-6$ dB dataset. (**a**) Test results of the WKN-X on the $-6$ dB dataset. (**b**) Test results of the WKN-Y on the $-6$ dB dataset. (**c**) Test results of modified-WKN on the $-6$ dB dataset. Class 0: normal state. Class 1: incipient unbalance. Class 2: mild unbalance. Class 3: moderate unbalance. Class 4: severe unbalance.

During the training process, the Adam optimizer was used with a learning rate of 0.001 for all models. The average of the five training results was taken as the final result to minimize the effect of randomness. The training results after 30 epochs are shown in Table 4. The t-distributed stochastic neighborhood embedding (t-SNE) of the model classification results is shown in Fig. 7. By observing the data in Table 4, it can be concluded that the performance of the modified WKN is excellent in all four datasets with different signal-to-noise ratios. Specifically, the accuracy of modified-WKN is more than 17 percentage points higher than WKN on the -4 dB and -6 dB datasets. In addition, the stability of WKN-X and WKN-Y is relatively lower than the modified WKN.

**Table 4:** The accuracy of WKN and modified-WKN under different noise conditions.

Method	0 dB	$-2$ dB	$-4$ dB	$-6$ dB
WKN-Y	80.56%±1.76%	80.69%±2.39%	79.86%±3.22%	74.14%±3.38%
WKN-X	81.95%±2.31%	80.76%±3.03%	79.86%±2.52%	77.28%±1.41%
Modified-WKN	99.65%±0.60%	99.10%±1.19%	97.92%±1.15%	94.65%±1.11%

**Figure 8:**
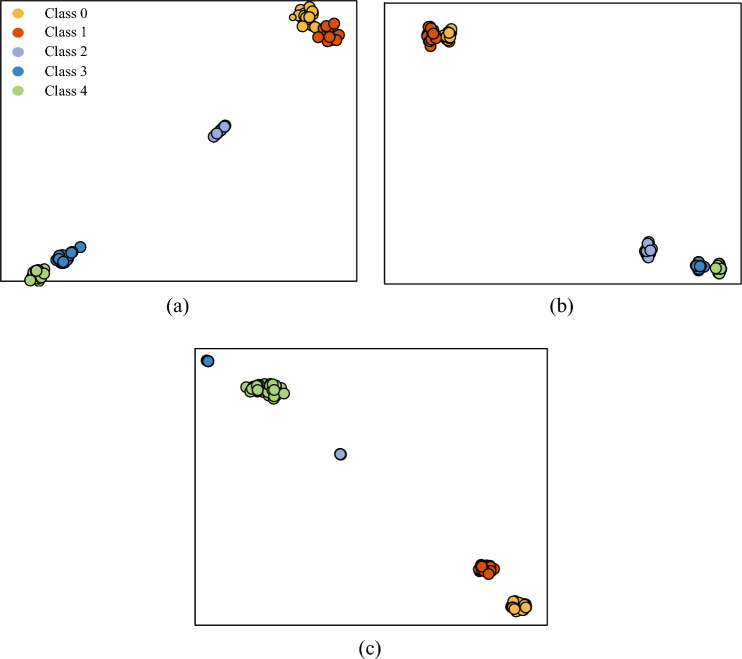
t-SNE visualization of modified-WKN at 1200 rpm, 1600 rpm, and 1800 rpm on the $-6$ dB dataset. (**a**) Test results of the 1200 rpm dataset. (**b**) Test results of the 1600 rpm dataset. (**c**) Test results of the 1800 rpm dataset. Class 0: normal state. Class 1: incipient unbalance. Class 2: mild unbalance. Class 3: moderate unbalance. Class 4: severe unbalance.

In order to observe the classification ability of the modified-WKN in a more detailed way, 1800 rpm, 1600 rpm and 1200 rpm were selected as the test data of the modified-WKN from 300 test sets. Subsequently, the output of the model was visualized using t-SNE. The classification results of modified-WKN are shown in Fig. 8. Although the modified WKN outperforms the WKN, its accuracy is still unsatisfactory in -4 dB and -6 dB noise environments. As shown in Fig. 8, the modified-WKN is unable to capture the weak incipient unbalance fault features hidden in the noise, resulting in more misclassifications of Class 0 and Class 1. Therefore, in order to minimize the interference of noise on the incipient weak unbalance signal, RIME-VMD and improved WKN based fault diagnosis is further investigated in the next subsection.

### Results based on the RIME-VMD and modified-WKN

In order to verify the effectiveness of the RIME-VMD method, the RIME-VMD method is compared with the VMD method and the GWO-VMD method (Gray Wolf Algorithm, GWO) in this paper. The detailed procedure of GWO-VMD can be found in the Ref.^28^. The detailed procedure of this experiment is as follows.

**Figure 9:**
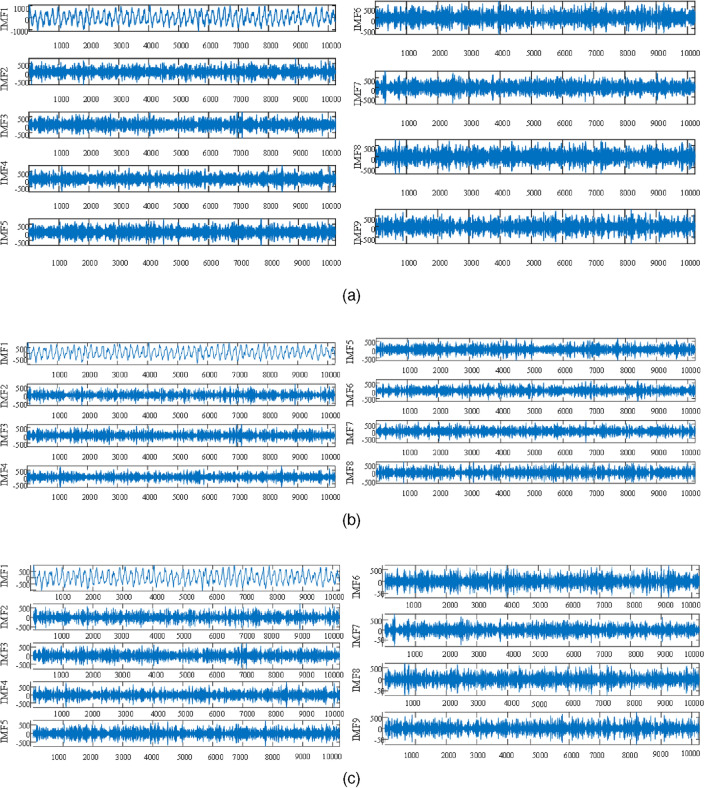
The results of the incipient unbalanced fault decomposition based on the RIME-VMD method, the GWO-VMD method and the VMD method. (**a**) is the decomposition result of the RIME-VMD method; (**b**) is the decomposition result of the GWO-VMD method; (**c**) is the decomposition result of the VMD method.

In the RIME-VMD method and the GWO-VMD method, the search range of the decomposition modulus number *K* is set as the range of [3, 10]; the search range of the penalty factor $\alpha$ is set as the range of [100, 2500]; the search agent quantity is set as 20. In the VMD method, the values of the number of decomposition layers *K* and the penalty factor $\alpha$ are set according to the signal processing experience. The X direction vibration signal depicted in Fig. 6a is used as the analysis sample in this experiment. After several rounds of iterations, the optimal parameter combination $(K,\alpha ) = (9, 560)$ is found by the RIME algorithm, which indicates that the number of decomposition layers *K* is 9, and the penalty factor $\alpha$ is 560. The GWO algorithm finds the optimal parameter combination $(K,\alpha ) = (8,2383)$, namely, the number of decomposition layers *K* is 8, and the penalty factor $\alpha$ is 2383. The value of the parameter combination $(K,\alpha )$ is set to (9, 1472) in the VMD method. The decomposition results of RIME-VMD, GWO-VMD, and VMD are shown in Fig. 9.

**Figure 10:**
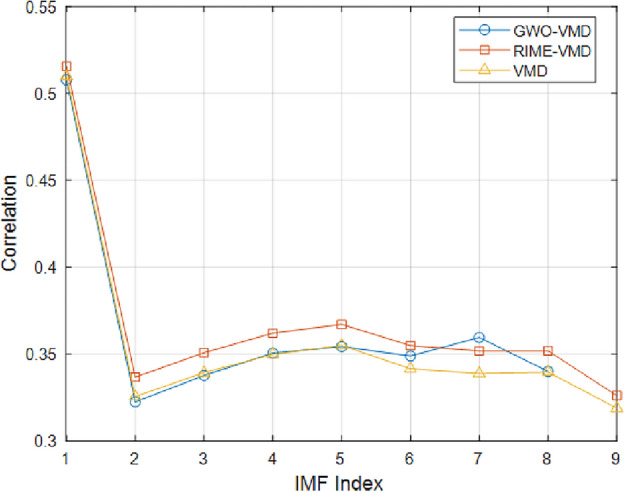
Pearson correlation coefficient of IMF components.

**Figure 11:**
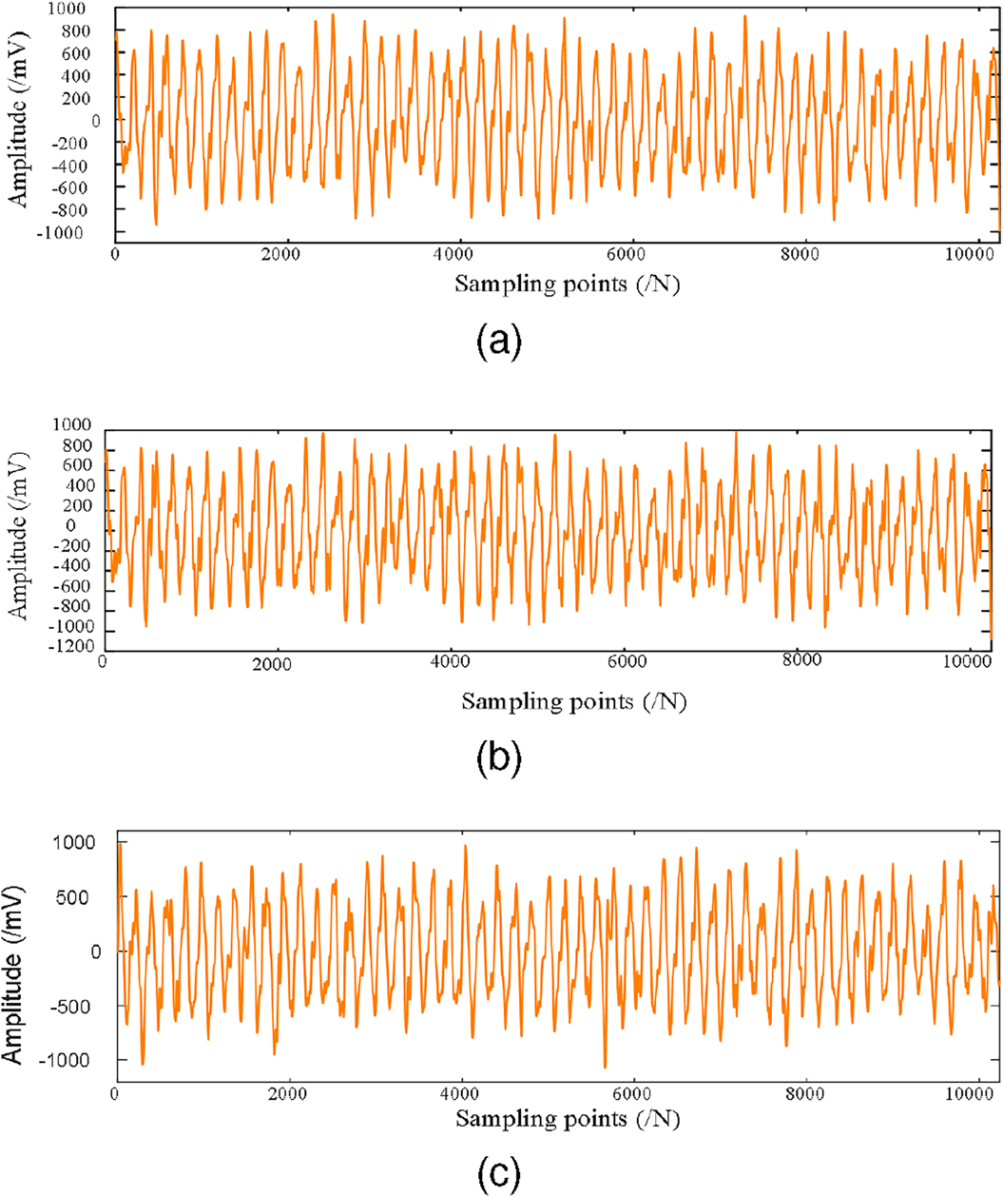
Reconstructed signal. (**a**) shows the reconstructed signal based on the one obtained with the RIME-VMD method; (**b**) shows the reconstructed signal based on the GWO-VMD method; (**c**) shows the reconstructed signal based on the VMD method.

The PCC reflects the degree of correlation between two variables. The higher the value of the PCC, the higher the correlation between the two variables. As shown in Table 5, the correlation coefficients were categorized as no correlation, weak correlation, moderate correlation, significant correlation and strong correlation^35^. In this experiment, the threshold for the PCC is established at 0.4. If the calculated correlation coefficient of the IMF component is higher than 0.4, the IMF component will be used for signal reconstruction. On the contrary, if the IMF component correlation coefficient is lower than 0.4, the IMF component will be discarded for signal noise reduction. The correlation coefficients of each IMF component were calculated using PCC, and the calculated results are shown in Fig. 10. The IMF components whose correlation coefficients are greater than the PCC threshold are selected for reconstructing the signal, and the reconstructed signal is shown in Fig. 11.

**Table 5:** Correlation coefficients and correlation.

Correlation coefficient	Relativity
$|r| \leqslant 0.2$	No correlation
$0.2<|r| \leqslant 0.4$	Weak correlation
$0.4<|r| \leqslant 0.6$	Moderate correlation
$0.6<|r| \leqslant 0.8$	Significant correlation
$0.8<|r| \leqslant 1$	Strong correlation

The training results for the reconstructed $-4$ dB and $-6$ dB datasets are shown in Table 6. Among them, WKN-X, WKN-Y, and Modified-WKN are the training results of the reconstructed dataset based on the RIME-VMD method. GWO-Modified-WKN is the training result of the reconstructed dataset based on the GWO-VMD method. VMD-Modified-WKN is the training result of the reconstructed dataset based on the VMD method. The diagnostic accuracies of both modified-WKN and WKN were significantly improved after extracting the unbalance fault information using the RIME-VMD method. However, it is of concern that the diagnostic performance of WKN still lags behind modified-WKN. Specifically, the modified-WKN fault diagnosis model achieves 99.03% and 99.45% accuracy on the reconstructed $-4$ dB and $-6$ dB datasets, respectively. In comparison, the accuracy of WKN-X and WKN-Y is 95.28% and 90.07% in the $-4$ dB dataset and 94.38% and 89.72% in the $-6$ dB dataset, respectively. From the perspective of model stability, the WKN exhibits significant fluctuations in diagnostic accuracy, showing poorer stability than the modified-WKN.

**Figure 12:**
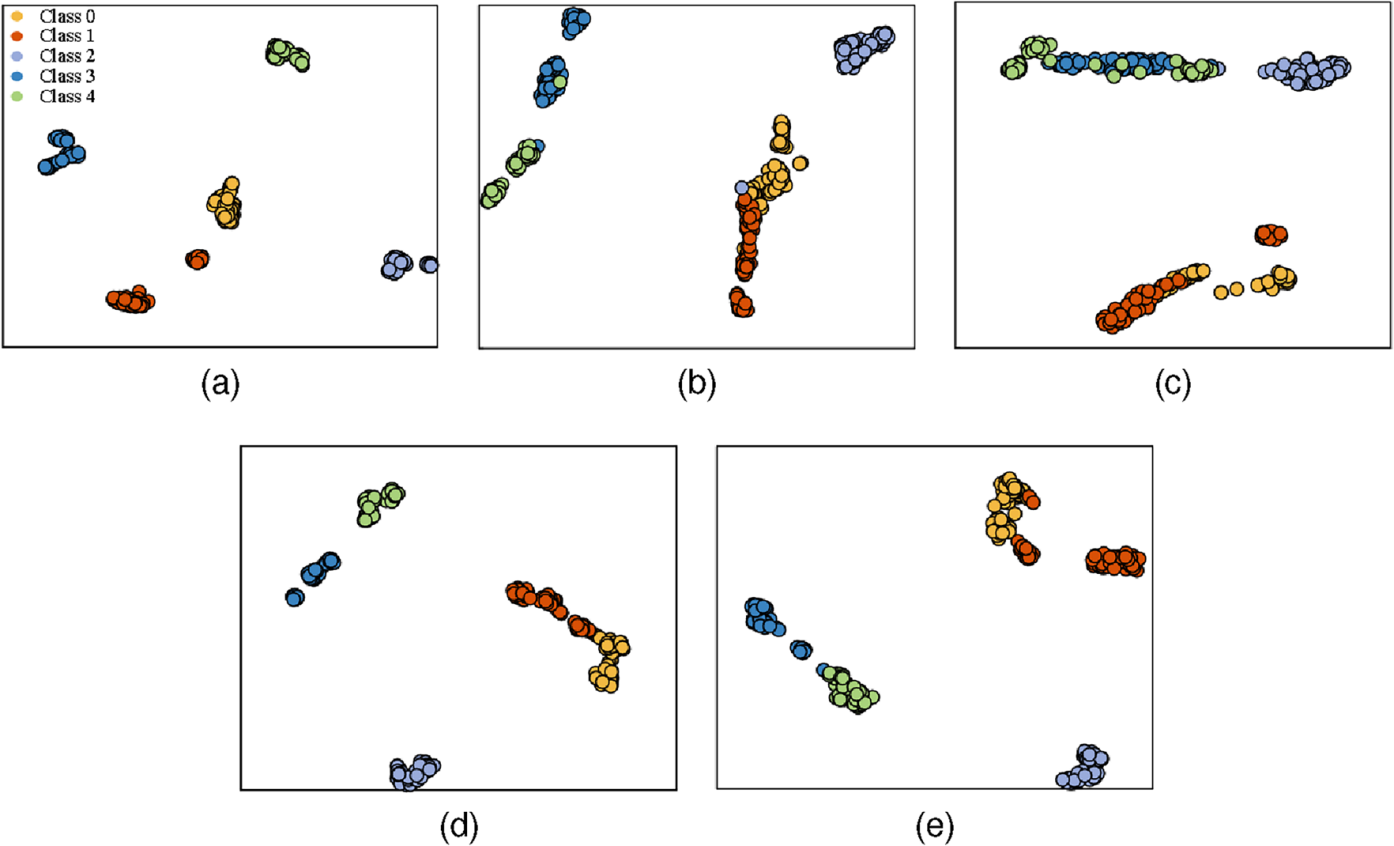
t-SNE visualization of the WKN the and modified-WKN Classification results on the $-6$ dB reconstructed dataset. (**a**) Test results of the modified-WKN based on the $-6$ dB reconstructed dataset. (**b**) Test results of the WKN-X based on the $-6$ dB reconstructed dataset. (**c**) Test results of the WKN-Y based on the $-6$ dB reconstructed dataset. (**d**) Test results of the GWO-Modified-WKN based on the $-6$ dB reconstructed dataset. (**e**) Test results of the VMD-Modified-WKN based on the $-6$ dB reconstructed dataset. Class 0: normal state. Class 1: incipient unbalance. Class 2: mild unbalance. Class 3: moderate unbalance. Class 4: severe unbalance.

In view of extracting unbalance fault information methods, the RIME-VMD, the GWO-VMD, and the VMD methods all improve the diagnostic performance of Modified-WKN. However, among them, the diagnostic performance of Modified-WKN based on the RIME-VMD method is better. In addition, as shown in Fig. 12, GWO-Modified-WKN, VMD-Modified-WKN, and WKN based on single-sensor data (WKN-Y, WKN-X) are not able to effectively differentiate between the normal states and the incipient unbalance states.

**Table 6:** Accuracy of WKN and modified-WKN on reconstructed datasets.

Method	$-4$ dB	$-6$ dB
WKN-Y	90.07%±2.02%	89.72%±1.09%
WKN-X	95.28%±1.99%	94.38%±2.02%
Modified-WKN	99.03%±0.76%	99.45%±0.39%
GWO-modified-WKN	98.27%±0.82%	98.08%±0.45%
VMD-modified-WKN	98.78%±0.83%	98.69%±0.98%

**Figure 13:**
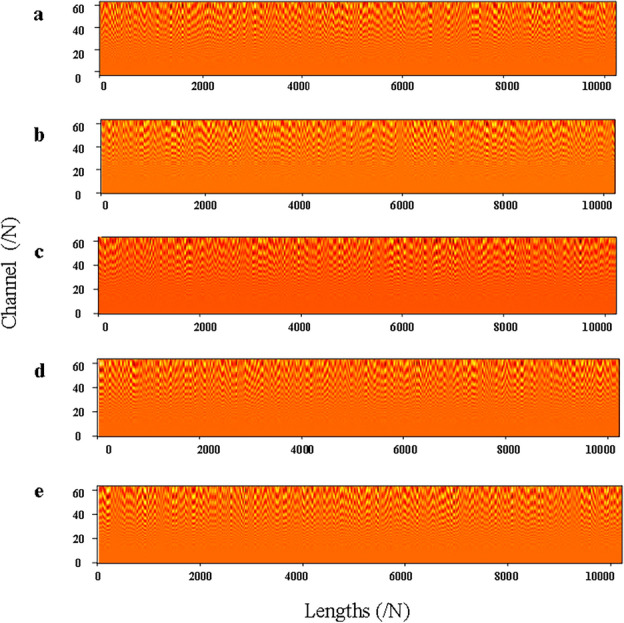
Visualization of the output features of CWConv layer in different states based on the $-6$ dB dataset.

**Figure 14:**
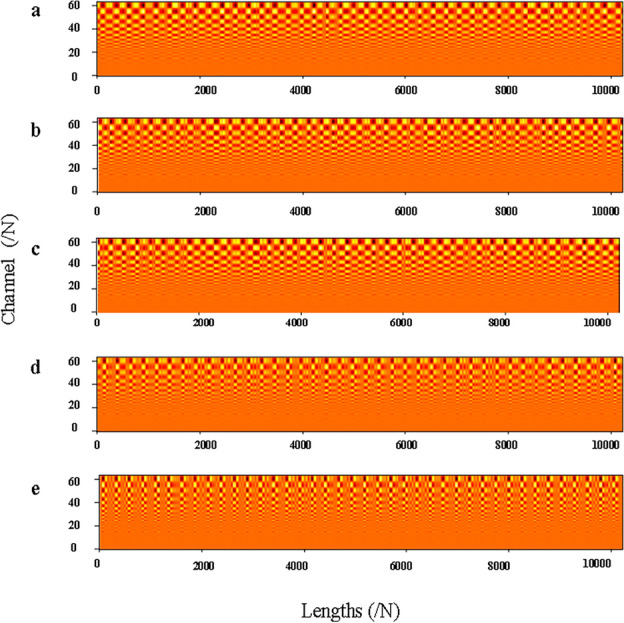
Visualization of the output features of CWConv layer in different states based on the $-6$ dB dataset with the RIME-VMD method.

The physical meaning of WKN is reflected in the output features of the CWCov layer. This means that the interpretability of WKN can be expressed through the visualization of the feature maps of the CWConv layer. In addition, the degree of energy concentration on the feature map can also be used to observe the impact of noise on the model’s feature extraction. Therefore, in this paper, the feature maps of CWConv layers in different health states are visualized. The feature visualization on the $-6$ dB rotor unbalance fault data is shown in Fig. 13. The visualization of the features on the reconstructed$-6$ dB dataset based on the RIME-VMD method is shown in Fig. 14. In Figs. 13 and 14, the feature-length is represented by the horizontal axis, and the number of channels is represented by the vertical axis. Where (a) shows the normal state, (b) shows the incipient unbalance state, (c) shows the mild unbalance state, (d) shows the moderate unbalance state, and (e) shows the severe unbalance state. From Fig. 13, it can be observed that the concentration of energy in the feature map is poor. The reason is that the presence of noise interference causes a large number of fault features to be overwhelmed by noise. After extracting the most relevant fault features using the RIME-VMD method, the energy of the output feature map of the CWConv layer is very concentrated.

## Conclusion

A new method of small unbalance fault diagnosis based on the RIME-VMD and modified-WKN is proposed in this paper. With the main contributions of the proposed method, the most relevent rotor fault information is extracted through the RIME-VMD, and the small incipient fault can be effectively detected by implementing the multi-head convolution and multi-scale convolution structure. According to the comparision of experiment results, it is demonstrated that the proposed method is more sensitive to the small incipient unbalance faults under the condition of noise. In the future study, the inner dynamics information of different rotor faults can can be ininvestigated combining with the proposed method to further increase the diagnosis performance and applicability.

## Data Availability

The data presented in this study are available on request from the corresponding authors.
